# Editorial: Preprocedural Computational Modeling — One More Step Toward Precision Medicine in Transcatheter Aortic Valve Replacement?

**DOI:** 10.1016/j.shj.2022.100045

**Published:** 2022-06-07

**Authors:** Ole De Backer, Maarten Vanhaverbeke

**Affiliations:** The Heart Centre, Rigshospitalet, Copenhagen University Hospital, Copenhagen, Denmark

In a decade’s time, transcatheter aortic valve replacement (TAVR) has evolved from a treatment option reserved for patients with severe, symptomatic aortic stenosis (AS) with a high surgical risk profile to a standard and preferred treatment option for nearly all severe AS patients ≥75 years regardless of the degree of surgical risk.^1^ In this same period, technological improvements in transcatheter heart valve (THV) systems have led to a significant increase in efficacy and safety of TAVR. Newer-generation devices with external sealing skirts have addressed the issue of paravalvular regurgitation (PVR), and delivery systems with a smaller insertion profile allow for a safe transfemoral approach. Together with improved operator experience and new implantation techniques, procedural outcomes of TAVR have improved steadily. In this context, use of the cusp overlap technique for implantation of self-expanding THVs has been reported to reduce permanent pacemaker implantation (PPI).^2^

Another important and underestimated contributor to the success of TAVR has been the improved preprocedural imaging and planning based on a standardized cardiac computed tomography analysis. Based on the patient-specific anatomical characteristics, a more patient-tailored TAVR treatment can be prepared for and performed.^3^ However, one of the remaining difficulties and limitations is that the exact device-host interaction cannot always be easily predicted. As a result, advanced computer technologies based on machine learning, pattern-recognition, and artificial intelligence have been developed over the past few years, generating computational models which can bring us one step closer toward ‘precision medicine’ and help the operator in preparing for the best possible TAVR outcomes.

In this issue of Structural Heart, Dowling et al.^4^ report on the performance of computed tomography–based computer-simulated contact pressure calculations to predict major conduction disturbances after TAVR. In a correct, retrospectively designed study in 80 patients, the authors modeled the contact pressure index (CPI) and maximal contact pressure (CPmax) of the implanted THV and measured at a predefined region of interest containing the atrioventricular conduction system. A prior study with early-generation self-expanding THVs had shown that computer simulations were able to identify patients at risk for major conduction disturbance and that the optimal thresholds for predicting conduction disturbance were a CPI ≥14% and a CPmax ≥0.39 MPa.^5^ However, with the introduction of newer-generation THVs with an outer sealing skirt, this model needed to be revalidated.

In this new study, Dowling et al.^4^ could confirm that the computer simulations could predict major conduction disturbances with acceptable diagnostic performance when simulating current-generation devices. Interestingly, the optimal threshold of the CPI to predict major conduction disturbance was slightly higher (CPI ≥20%) than previously reported; this might be attributable to differences in the design of the current-generation Evolut PRO THV, which has an outer pericardial sealing skirt. Furthermore, the mean CPI measured in this study (20%) was lower than that in prior studies, possibly reflecting an ability of the operators to implant THVs systematically higher in current practice. The optimal CPmax for predicting major conduction disturbance (≥0.40 MPa) was similar to that previously reported, confirming the important role that this variable plays in predicting conduction disturbance. The computer simulations could also identify patients at risk for PPI—this risk was highest for patients who had both a CPI ≥20% and a CPmax ≥0.40 MPa. The study could also confirm that implantation depth was predictive of major conduction disturbance; however, it did not find membranous septum length nor calcium volume to be predictive of this outcome.

Despite these promising results reported by Dowling et al., it is important to realize that these computer simulations did not demonstrate perfect diagnostic accuracy (area under the curve <0.80). Several factors could be responsible for this observation: (1) anatomical variations in the atrioventricular bundle and proximal left bundle branch vs. oversimplification in the model, (2) pre-existing and even masked conduction disturbances which are not modeled in the predictive models, and (3) limitations of the computer models with regard to the use of predilatation and postdilatation and/or repositioning(s) of the THV. Similar limitations are applicable to the computational models predicting PVR after TAVR.^6^ The authors also sought to examine whether computer simulations could identify patients at risk for prolonged hospitalization and long-term adverse clinical outcomes.^4^ However, the further the studied outcomes are ‘distanced’ from the pure host-device interaction modeling and the more other variables also impact these clinical outcomes, the less diagnostic accuracy can be expected of these computer simulations.

In daily clinical practice, the predictive computational models for PVR and PPI could be utilized by the TAVR operator to further improve and fine-tune the preprocedural planning, aiming for the best possible patient-tailored decision on THV type, size, and implant depth and, ultimately, the best possible clinical outcome.

Whether computational modeling will become a new standard for all TAVR procedures and/or other structural heart procedures—such as transcatheter mitral and tricuspid valve interventions, percutaneous left atrial appendage closure, etc.—is difficult to foresee and will depend on the availability, ease of use, and cost of these additional imaging tools. However, it is very plausible that this technology and a higher degree of ‘precision medicine’ are desirable when treating more complex and younger AS patients. There is already good evidence that patient-specific computer simulations of THV sizing and positioning improve clinical outcomes in bicuspid aortic valves.^7^ When treating younger patients with TAVR, also other outcomes such as obtaining the lowest possible transprosthetic gradient, commissural alignment, and preserved coronary access become increasingly important aspects that determine procedural success. Furthermore, computational modeling may also play and claim a role in the planning and decision-making process for ‘lifetime management’ of younger patients with severe AS, helping to understand and simulate which future valve-in-valve combinations and solutions may give the patient the best possible clinical outcome at the long term.

In summary, we can conclude that computational modeling is a promising new technology which may bring us a step closer to ‘precision medicine’ in TAVR and other structural heart procedures. We are only seeing the beginning of the full potential of these more advanced imaging technologies supporting complex heart interventions, as logical next steps would be to have the computational models being calculated in ‘real time’ and being integrated in image fusion technologies with other imaging modalities. This once ‘far-distant’ future is not that distant any longer.

## Funding

The authors have no funding to report.

## Disclosure statement

The authors report no conflict of interest.
